# The Story of the Insane from Year to Year

**Published:** 1900-01-27

**Authors:** 


### THE STORY OF THE INSANE FROM YEAR TO YEAR.

Twelfth Series.

### KENT COUNTY ASYLUM AT MAIDSTONE.

This asylum contained 1,624 patients at the beginning of the year, and 1,533 at the end of it, but this decrease has been owing to the transfer of patients to the other Kent asylum at Chartham. There were 372 first admissions, 54 readmissions, 134 recoveries, and 180 deaths. The recoveries were 31·6 per cent. of the admissions, and the deaths were 11·3 per cent. of the average number resident. Cerebral and spinal diseases account for 41 of the deaths, bronchitis for 4, pneumonia for 24, and consumption for 49. Colitis was the cause in 2, tuberculosis in 4, and influenza in 3. Dr. Pritchard says that the "general health of the patients has been good," but the mortality from lung disease strikes us as being excessive, and as needing some explanation. At the Barming Heath Asylum, with an average number of 1,588 patients, there were 77 deaths from these diseases, whereas in the Chartham Asylum, with an average number of 1,027 patients, there were only 27 deaths from lung diseases. In 26 of the admissions the insanity was caused by moral causes, in 35 by intemperance in drink, and in 71 by hereditary influence. The average weekly rate of maintenance was 9s. 1¾d. per head.

### WILTS COUNTY ASYLUM, DEVIZES.

On January 1st, 1898, there were 825 patients in residence, and by the end of December the number had risen to 851. There were 133 first admissions, 27 readmissions, 47 recoveries, 10 discharged relieved, and 73 deaths. The recoveries stand at 29·3 per cent. of the admissions, and the deaths at 8·6 on the average number resident. Both of these percentages are low. Cerebral and spinal diseases account for 24 of the deaths, pneumonia for 6, bronchitis for 2, and consumption for 6. Table X. shows us that intemperance in drink caused the insanity in 18 of the admissions and hereditary influence in 48. Mr. Bowes finds that the mortality from consumption in the Devizes Asylum is a diminishing one, and he suggests that this diminution may be owing to better sanitary arrangements, absence of overcrowding, and the use of dry, polished floors instead of the old wet, scrubbed ones. No doubt all these help, but open air by day and open windows by night will do more still. Last year Mr. Bowes had prolonged sick leave, and we are pleased to learn that he returned much better from his holiday. The visitors say "that the management of the asylum by the medical superintendent has their highest approbation." The weekly cost of maintenance was 9s. 3¾d. per head.

### NORWICH CITY ASYLUM, HELLESDON.

An increase of 16 patients during the year would not mean much in most asylums, but here it means a good deal, as the total number is only 302. There were 82 first admissions, 15 readmissions, 23 recoveries, 13 discharged relieved, and 41 deaths. The percentage on recoveries was only 25·84. This is considerably below the average of the Norwich Asylum, and Dr. Harris says the percentage would have been 57·5 had he included those previously admitted. This probably means that the low percentage has been obtained by reckoning only those cases admitted during the year. If so, it is not the usual way in asylums. The death-rate is 13·71 per cent. on the average number resident. This is nearly 4 per cent. above the average. Of these deaths 20 were caused by cerebral and spinal diseases, 4 by consumption, and 1 by acute tuberculosis. Of the year's admissions 11 owed their insanity to moral causes—domestic trouble, &c., 12 to intemperance in drink, and 17 to hereditary influence. Dr. Harris has always been interested in the family history of his patients, and he points out "that in each successive generation of the insane insanity often showed itself earlier in life than in members of the previous generation, and so in time direct insane issue died out." We wish we could think so, but we cannot accept this crumb of consolation, for undoubtedly many of these of the younger generation are discharged "recovered," as it is called, and are no sooner out of the asylum than they marry and are given in marriage. Indeed, Dr. Harris himself adds that the new cases of insanity keep pace with the increase of the city population; and so they will so long as he cures 57 per cent. or even 25 per cent. of the admissions. We are glad to note that only 12 post-mortem examinations were made during the year, and we venture to hope that Dr. Harris will not copy the examples elsewhere set, but that he will confine these examinations to cases wherein the diagnosis of the disease was doubtful, or wherein there was good reason to believe that some useful lesson would be learnt, and that in all cases he will obtain the relatives' consent. The visitors place on record their "great satisfaction at the long-continued and unbroken reliance" they repose in their medical superintendent. The weekly cost of maintenance is 9s. 3½d. per head.

### THE EDINBURGH ROYAL ASYLUM.

On January 1st, 1898, there were in residence 888 patients, and in addition to this number 11 were absent on probation. On December 31st the number had risen to 945. There were 347 first admissions, 105 readmissions, 163 recoveries, 94 discharged relieved, and 103 deaths. Calculated on the admissions, the recoveries were 36 per cent., and on the average number resident the deaths were 7·6 per cent. Of the deaths during 1898, 49 were caused by cerebral and spinal diseases, 12 by consumption, and 6 by pneumonia. In 41 of the admissions the insanity was due to moral causes, in 103 to intemperance in drink, in 125 to hereditary influences, and in 208 the cause was unknown. Such a very high percentage of "unknowns" is most unusual nowadays, and it militates against any reliable inference being drawn from the statistics. Dr. Clouston thinks that the number of admissions will increase for many years to come—not because there are more lunatics, but because society is more and more realising the benefits which the insane derive from hospital treatment. This fact does explain part, at least, of the increase in the numbers of the insane, that is, of the registered insane. When a new case of mental disease is being examined, Dr. Clouston asks himself the question, "Could this have been altogether prevented, and how?" And he answers that "it could not have been done in most cases, for there had existed from the first in the patient's brain an *hereditary* predisposition to a nervous instability, if not to insanity, that had been roused into actual disease by events in his physiological life, or his circumstances, or his environment, over which he or other had little control." We believe this sentence to be as full of truth as it is wanting in consolation; and we have italicised the word "hereditary," as it points to the irresistible conclusion that such people ought never to have been brought into the world at all; but we have not much hope that either Dr. Clouston or ourselves will obtain a hearing until public opinion is sufficiently educated to see that the marriage of the parents was an awful crime. As to the drink cases, Dr. Clouston thinks that many of the 103 might have saved themselves, and he adds that a terrible penalty has been exacted from them. Doubtless it has, but, alas! the penalty falls far beyond the drunkard himself. Of the total number of patients 394 are private cases. The rate of maintenance for paupers is £31 per head per annum. A table showing the various sums at which private patients are admitted would be most useful for comparison.

### IPSWICH BOROUGH ASYLUM.

From this, the twenty-eighth annual report, we learn that this asylum had in it on the last day of 1870 only 108 patients, and that it now has 317, having trebled its population since its opening. During 1898 there were 123 first admissions, 7 readmissions, 33 recoveries, and 38 deaths. The recoveries stand at 25·3 per cent. of the admissions, and the deaths are stated to be 10·7 per cent. on the average number resident; but we do not see how this is arrived at, as the death-rate on the men's side is given as 16·1, and that on the women's side as 9·4. Probably the "10" is a misprint for "12." Of the year's deaths, cerebral and spinal diseases account for 16, consumption for 6, and congestion of the lungs for 1. The usual moral causes account for the insanity of 17 of the admissions, while physical causes are responsible for 117, and of the latter 14 were intemperance in drink and 18 hereditary influence. The committee say that "the management of the asylum, and the conduct of the officers and attendants and the care of the patients have been satisfactory." The weekly cost of maintenance is 8s. 9d. per head.

### THE BERKSHIRE COUNTY ASYLUM AT MOULSFORD.

On the first day of 1898 this asylum contained 623 patients, and on the last day of December the number had increased to 638. There were 107 first admissions, 16 readmissions, 39 recoveries, 11 discharged relieved, and 52 deaths. The recoveries stand at 29·7 per cent. of the admissions, transfers being included, and the deaths at 8·22 per cent. of the average number resident. Of the deaths during the year 19 were caused by cerebral and spinal diseases, 14 by consumption, 4 by pneumonia, 2 by bronchitis, and 2 by enteric fever. Table X. shows that only 2 of the year's admissions had their illness caused by moral causes; but in 47, heredity was the cause, either alone or combined with other causes. General paralysis figures as a cause in 12 cases, but it is the insanity itself and not the cause. Dr. Murdoch mentions the case of a criminal lunatic who had been in Broadmoor for committing murder, and who was transferred to the Berkshire Asylum. "It is much to be desired," continues Dr. Murdoch, "that some further State accommodation should be provided for the detention of criminal patients, in order to avoid the association of this class with the insane confined in public asylums, which is much to be deprecated." For twenty years and more this cry has gone up from almost every asylum in England, and almost since its origin the hospital has advocated the building of another criminal asylum, but so far Government has turned a deaf ear to the cry. When speaking of heredity, Dr. Murdoch tells us two women, congenitally deficient in mind, were married, and had each four children and were then sent to the asylums, and he asks, "is it to be wondered at that such an increasing tax is put upon the ratepayers for the providing of asylum accommodation when the marriage of such persons is so prevalent." No, it is not to be wondered at, and if Dr. Murdoch be a constant reader of this journal he will remember that we have referred to the matter over and over again. The mortality from consumption is unusually high, but it would seem that in six instances the disease existed on admission, and in four there was a strong tendency to it. Nevertheless, we should like to know what system of ventilation is in use in the dormitories, and how many hours the patients spend out of doors daily. Enteric fever made its appearance in May and lasted until November. The origin of the disease could not be discovered, and we have often found this to be the case in these asylum outbreaks. Here the drainage, vegetables, milk, and the water supply were known to be excellent. Altogether there were six cases and four deaths, two of the latter being nurses. The weekly cost of maintenance was 7s. 10½d. per head.

### THE KENT COUNTY ASYLUM AT CHARTHAM.

ON January 1st, 1898, this asylum contained 944 patients, and on December 31st had risen to 1,125. This increase, as already mentioned when speaking of the Barming Heath Asylum, was in great part owing to the transference of patients from the latter. There were 379 admissions, 74 recoveries, 6 discharged relieved, and 98 deaths. The recoveries stand at 37 per cent. of the admissions, and the deaths at 9 per cent. on the average number resident. Of the year's deaths 41 were caused by cerebral and spinal diseases, 8 by pneumonia, 1 by bronchitis, 13 by consumption, and 2 by colitis. Various moral causes are credited with causing the insanity in 45 of the admissions, intemperance in drink in 35, and hereditary influence in 76. During the year additions for 300 patients were opened, and there are now, or were at the close of 1898, vacancies for 80 patients. The drainage system was examined, and, Dr. Fitzgerald says, "resulted in wholesale condemnation," and is to be altered and carried out on a system fulfilling "the requirements of modern sanitary methods." The Chartham Asylum is only 25 years old, and yet its sanitary arrangements already need remodelling. We can only guess what state of things exists in the older county asylums. The weekly rate of maintenance is a little over 10s. a head.

### THE WEST RIDING ASYLUM AT MENSTON.

AT this asylum the numbers were almost stationary during the year 1898. On December 31st there were 1,530 patients in residence, being only 4 in advance of the numbers on January 1st. There were 482 first admissions, 90 re-admissions, 223 recoveries, 22 discharged relieved, and 176 deaths. The recoveries, calculated on the admissions, were 38·98 per cent., and the deaths, calculated on the average number resident, were 11·34 per cent. The recoveries are quite up to the average, and the deaths are somewhat higher than the average of kindred institutions. Seventy of the deaths were caused by cerebral and spinal diseases, 11 by pneumonia, 17 by consumption, and 5 by enteritis. The usual moral causes figure in the year's admissions. We notice that 60 owed their insanity to domestic trouble, 77 to mental anxiety, and 17 to religion. Among the physical causes, intemperance in drink and hereditary tendency occupy their usual prominent places; the former accounting for 75, and the latter for 121. There were upwards of 30 cases of colitis, and, as already said, 5 deaths. Dr. McDowall thinks the disease was imported by a recently-admitted patient. It is a most unfortunate occurrence, as there is such a strong probability of recrudescence of the disease when it is least expected. The Commissioners in Lunacy advise the erection of a chapel, but when we learn that 580 patients were able to attend service in the hall at much less trouble to themselves and the staff than if they had a detached chapel, it is not easy to see any reason why a cost of several thousand pounds should be incurred and less than nothing obtained by it. Dr. McDowall has now four assistant medical officers, and one of these is a lady. There is also a qualified lady doctor as clinical clerk. Assuredly Dr. McDowall was well advised in making these appointments. The committee "record their appreciation of the constant care shown by the medical superintendent and his assistants." The weekly cost of maintenance was 8s. 11¼d. per head.

